# Giant renal angiomyolipoma: A case report

**DOI:** 10.1016/j.ijscr.2023.108538

**Published:** 2023-07-20

**Authors:** Tilahun Deresse, Mandante Bogale, Dawit Alemayehu, Megbar Dessalegn, Marta Seid

**Affiliations:** a Department of Surgery, School of Medicine, Debre Berhan University, 445, Ethiopia; b Department of Surgery, School of Medicine, Debre Markos University, 269, Ethiopia; c Department of Surgery, School of Medicine, Saint Paul Hospital Millennium Medical College, Addis Ababa 1271, Ethiopia

**Keywords:** Giant angiomyolipoma, Retroperitoneal mass, Case report

## Abstract

**Introduction:**

Renal angiomyolipoma (AML), which is a rare solid kidney tumor with benign characteristics, also known as a renal hamartoma, can exhibit various clinical symptoms and severe consequences may arise if the lesion becomes large.

**Presentation of the case:**

A 58-year-old woman was admitted to a hospital, with general fatigue, abdominal swelling, and epigastric fullness. Upon examination, a large mass was palpated, which occupied almost the entire right abdomen. The abdominal computed tomography scan revealed a large right renal mass measuring 22 × 18 × 8 cm, which was exophytic and heterogeneous with a large fat component and an enhancing solid part. The tumor was successfully excised through a generous right subcostal incision with left-side extension. The total weight of the resected specimen was 2500 g, which appears to be the largest angiomyolipoma ever resected in Ethiopia.

**Discussion:**

Renal AML, a benign tumor derived from mesenchymal components, is sometimes referred to as a “hamartoma” due to its variable makeup. The most common complaints of patients with renal AML are lower back pain, hematuria, and physical finding of hypotension (shock), though patients with giant AML, as in this case, may also experience gastrointestinal symptoms due to the mass' compression.

**Conclusion:**

Although treatment options requiring contemporary medical technologies and skilled manpower are difficult to offer in set ups of resource-limited countries, such as the one we reported from, giant renal angiomyolipoma can be treated safely with open nephrectomy.

## Highlights

• Giant renal AML is a rare solid tumor.
• Most renal AMLs are asymptomatic and identified incidentally with imaging.
• Giant renal AMLs carry significant risk of bleeding.
• Partial or total nephrectomy is recommended treatment for giant AMLs.

## 1 Introduction

Angiomyolipoma constitutes a benign neoplasm of the kidney that encompasses a heterogeneous group of atypical blood vessels, smooth muscle, and adipose tissue. Although the preponderance of cases remain asymptomatic, certain tumors may elicit sudden abdominal pain or hypotension, due to substantial intra lesion hemorrhage or retroperitoneal invasion. The management of patients presenting with renal angiomyolipoma is largely determined by tumor size. Tumors with a diameter of less than four centimeters (cm) are typically asymptomatic and can be managed with close monitoring, whereas larger tumors require the use of one or more of the available treatment modalities, including radiofrequency ablation, transarterial embolization, and surgical intervention [1].

Herein, it is our intention to present a highly uncommon case in which the patient expressed dissatisfaction with abdominal fullness, ultimately leading to the diagnosis of a sizable renal angiomyolipoma. This medical anomaly was effectively resolved through the implementation of surgical excision. It is noteworthy that this particular case report adheres strictly to the esteemed SCARE criteria [2].

## 2 Case presentation

A 58-year-old female patient presented with general fatigue, abdominal swelling, epigastric fullness, early satiety, progressive bloating sensation, and reddish discoloration of urine lasting for six months before her presentation.

The abdominal swelling started as a small mass in the right flank area, which gradually increased to occupy almost the entire abdomen over months' period. Furthermore, the patient experienced a weight loss of about 6 kg, with a weight of 54 kg at presentation compared to 60 kg, six months prior. Upon examination, a smooth, round, soft, and painless mass was palpable on the entire abdomen, with the dominant portion localized on the right side. Further examination and investigations were conducted to determine the underlying cause of the symptoms presented by the patient, which included abdominal distension, weight loss, and reddish urine.

The complete blood count revealed a moderate level of anemia, indicated by a hemoglobin level of 8.0, while the other parameters fell within the normal range. Urine microscopy revealed microscopic hematuria, but renal function test results were unremarkable.

**Fig. 1:**
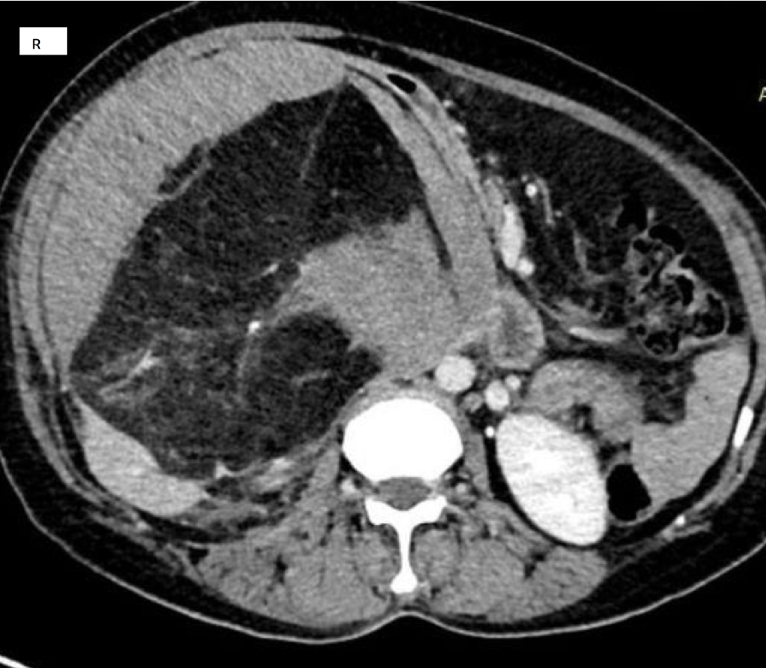
Abdominal CT scan, showing mixed density mass arising from the right kidney.

Following ultrasound examination, a hyper echoic mass measuring approximately 18 cm in size was identified in the right abdominal area, possibly originating from the right kidney. Additionally, computed tomography (CT) revealed a large right renal mass measuring 22 × 18 × 8 cm, which was exophytic and heterogeneous with a large fat component and an enhancing solid part (Fig. 1). There were no symptoms or radiological findings suggestive of tuberous sclerosis complex (TSC).

**Fig. 2:**
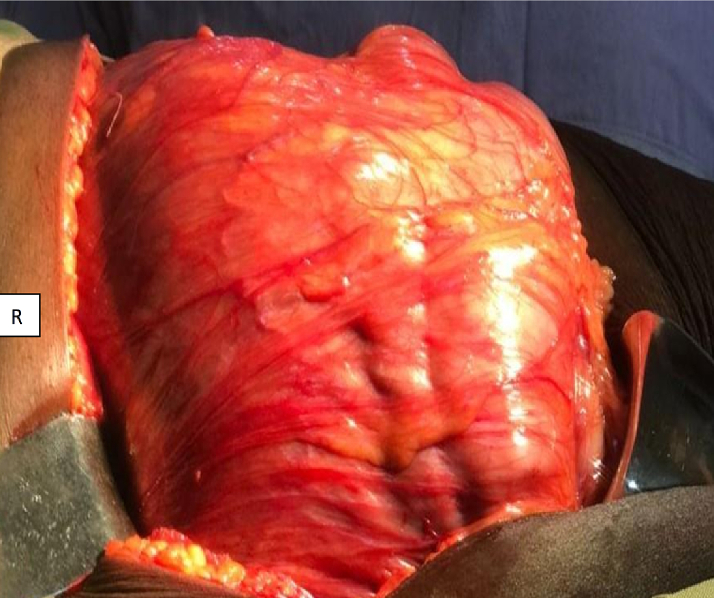
Exploratory laparatomy image showing, large mass occupying the right side of the abdomen, pushing the viscera to the left.

Exploratory laparotomy was performed via a subcostal incision on the right side. However, it was not possible to achieve full exposure through this incision; thus, we extended it to the left side, resulting in a chevron incision. The abdominal muscles were then divided using electrocautery, and the peritoneum was entered. As shown in Fig. 2, we found a large mass occupying the right side of the abdomen, pushing the bowel to the left. The posterolateral peritoneum was incised along the line of Toldt, and the ascending colon and duodenum were reflected medially to expose the renal vein and inferior vena cava and to develop the retroperitoneal space. The renal artery and vein were identified and double-ligated before division. The inferior pole of the kidney was dissected free and the ureter was identified, ligated, and divided. Then, the superior pole was dissected, the kidney with the mass was resected en mass, and hemostasis was secured. The estimated volume of intraoperative blood loss was 500 ml. The patient's recovery was uneventful and supportive care was provided through fluid infusion. The patient was discharged on the sixth postoperative day, with a postoperative hemoglobin level of 8.4 g/dl. On her first appointment, one week after discharge, she complained of mild wound site pain and her hemoglobin was 7.9 g/dl for which she was started and oral iron therapy. The patient was followed up monthly for the next 3 months and then every 3 months until she was discharged from follow-up at one year postoperative, and she was found to be disease-free on her last visit.

**Fig. 3:**
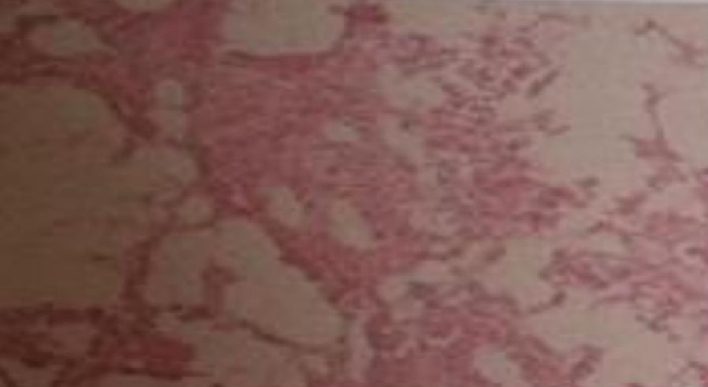
Histologic image showing proliferations of bland adipocytes admixed with cells having cigar shaped nuclei and eosinophilic cytoplasm and blood vessels, consistent with AML.

Histopathological analysis revealed proliferation of bland adipocytes intermixed with cells with eosinophilic cytoplasm and cigar-shaped nuclei (Fig. 3). The final diagnosis was based on the histopathological report, which indicated angiomyolipoma of the right kidney, and no immunohistochemical test was performed (not available in the setup).

## 3 Discussion

First documented in 1951, renal angiomyolipoma (AML) is a nonmalignant neoplasm originating from mesenchymal elements [3]. As a result of its heterogeneous composition, including adipose tissue, smooth muscle, and blood vessels, AML is also denoted as a “hamartoma” [4]. These growths are typically categorized into two subtypes, specifically within the context of Tuberous Sclerosis Complex (TSC) or as sporadic; the case under consideration pertains to the latter classification. According to previous reports [1,5], renal angiomyolipoma (AML) may exhibit an annual growth rate of up to 4 cm in its maximum dimension. The AML is considered “giant” when its diameter surpasses 10 cm. In the literature, there are few documented cases of giant renal AMLs [6], with Taneja and colleagues reporting the largest renal AML (39 × 25 × 9 cm) in 2013 [7].

This substantial tumor, possessing thick-walled blood vessels, is susceptible to vascular ruptures and hemorrhaging. The occurrence of compression symptoms and bleeding from rupture is heightened owing to the development of hemorrhagic aneurysms accompanying the expanding AML [8], which may necessitate prophylactic or therapeutic transarterial embolization [9]. The clinical indications that are most significant in indicating retroperitoneal bleeding, also known as Wunderlich's syndrome, include lower back pain, hematuria, and shock. These clinical symptoms are commonly the reason why patients with renal AML seek medical attention.

For tumors that are less than 4 cm in size, most cases are asymptomatic, and can be followed with conservative management [7], or radiofrequency ablation [10] can be administered. As the tumor grows larger, compression of the gastrointestinal tract may result in alimentary complaints, and a mass in the abdominal area may be easily detected by palpation. The echogenicity of the mass on ultrasound and its CT scan density resembling that of fat are indicative findings. When the renal AML's diameter exceeds 4 cm, especially in the case of large tumors with persistent hemorrhage or suspected malignancy, nephrectomy, either partial or total, is the favored treatment option [11,12]. Unfortunately, partial nephrectomy was not feasible in the present scenario, necessitating total nephrectomy. The histopathological examination of renal AML revealed the presence of normal/mature adipose tissue as the primary component [13]. HMB-45 positivity detected through immunohistochemical staining is a reliable tool to differentiate renal AML from other renal tumors [14], but it is not available in our facilities.

## 4 Conclusion

Although treatment options requiring contemporary medical technologies and skilled manpower are difficult to offer in set ups of resource-limited countries, such as the one we reported from, giant renal angiomyolipoma can be treated safely with open nephrectomy.

## Consent

Before data collection, written informed consent was acquired and the laboratory procedure was done with the essence of beneficence and data were kept confidential. The written informed consent was obtained from the patient for publication of this case report and accompanying images. A copy of written consent is available for review by the Editor-in-Chief of this journal on request.

## Ethical approval

The study is approved by the Research Review Committee of the institution. Before data collection, informed consent was acquired and the laboratory procedure was done with the essence of beneficence and data were kept confidential.

## Funding

There is no funding received for this work.

## Author contribution

Tilahun Deresse: Writing – original draft, review & editing.

Mandante Bogale: Writing – original draft, review & editing.

Dawit Alemayehu: Review & editing.

Megbar Dessalegn: Review & editing

Marta Seid: Review & editing

## Guarantor

Tilahun Deresse.

## Research registration number

Not applicable.

## Declaration of competing interest

There is no conflict of interest.
